# Effect of diet with or without exercise on abdominal fat in postmenopausal women – a randomised trial

**DOI:** 10.1186/s12889-019-6510-1

**Published:** 2019-02-11

**Authors:** Willemijn A. van Gemert, Petra H. Peeters, Anne M. May, Adriaan J. H. Doornbos, Sjoerd G. Elias, Job van der Palen, Wouter Veldhuis, Maaike Stapper, Jantine A. Schuit, Evelyn M. Monninkhof

**Affiliations:** 1 Department of Epidemiology, Julius Center for Health Sciences and Primary Care, University Medical Center Utrecht, STR 6.131, Universiteitsweg 100, 3584 CG Utrecht, the Netherlands; 2 Medisch Spectrum Twente Hospital, Department of Epidemiology, Enschede, the Netherlands; 3 Department of Research methodology, Measurement and Data analysis, University of Twente, Enschede, the Netherlands; 4 Department of Radiology, University Medical Center Utrecht, Utrecht, the Netherlands; 5 Division of Public Health and Health Care, National Institute for Public Health and the Environment, Bilthoven, the Netherlands; 6 Department of Health Sciences, EMGO Institute for Health and Care Research, VU University, Amsterdam, the Netherlands

**Keywords:** Weight loss, Intra-abdominal fat, Subcutaneous fat, Exercise

## Abstract

**Background:**

We assessed the effect of equivalent weight loss with or without exercise on (intra-) abdominal fat in postmenopausal women in the SHAPE-2 study.

**Methods:**

The SHAPE-2 study is a three-armed randomised controlled trial conducted in 2012–2013 in the Netherlands. Postmenopausal overweight women were randomized to a diet (*n* = 97), exercise plus diet (*n* = 98) or control group (*n* = 48). Both intervention groups aimed for equivalent weight loss (6–7%) following a calorie-restricted diet (diet group) or a partly supervised intensive exercise programme (4 h per week) combined with a small caloric restriction (exercise plus diet group). Outcomes after 16 weeks are amount and distribution of abdominal fat, measured by magnetic resonance imaging (MRI) with the use of the three-point IDEAL Dixon method.

**Results:**

The diet and exercise plus diet group lost 6.1 and 6.9% body weight, respectively. Compared to controls, subcutaneous and intra-abdominal fat reduced significantly with both diet (− 12.5% and − 12.0%) and exercise plus diet (− 16.0% and − 14.6%). Direct comparison between both interventions revealed that the reduction in subcutaneous fat was statistically significantly larger in the group that combined exercise with diet: an additional 10.6 cm^2^ (95%CI -18.7; − 2.4) was lost compared to the diet-only group. Intra-abdominal fat loss was not significantly larger in the exercise plus diet group (− 3.8 cm^2^, 95%CI -9.0; 1.3).

**Conclusions:**

We conclude that weight loss of 6–7% with diet or with exercise plus diet reduced both subcutaneous and intra-abdominal fat. Only subcutaneous fat statistically significantly reduced to a larger extent when exercise is combined with a small caloric restriction.

**Trial register:**

NCT01511276 (clinicaltrials.gov), prospectively registered.

**Electronic supplementary material:**

The online version of this article (10.1186/s12889-019-6510-1) contains supplementary material, which is available to authorized users.

## Background

Worldwide obesity has doubled since 1980 and in 2014 over 600 million people were found to be obese. [1] A high risk group for obesity and central obesity are postmenopausal women [2]. Menopause, aside from contributing factors as increasing age, lower energy expenditure and increased caloric intake, affects body composition by changing hormonal levels, fat distribution and insulin resistance, causing central obesity [2, 3]. Increased body fat and specifically abdominal fat has been associated with chronic diseases like cardiovascular diseases, type II diabetes, and higher all-cause mortality [4, 5]. Intra-abdominal fat, and not subcutaneous fat, is hypothesized to be most harmful for metabolic related diseases [6–8], and intra-abdominal fat volume is an independent predictor of insulin resistance, hypertension and myocardial infarction [9].

Intra-abdominal fat can be reduced by diet and/or exercise, but there is no general agreement on the most effective way to reduce fat in this specific area. In obese postmenopausal women, weight loss trials evaluating diet interventions with or without exercise, found that exercise is not more effective compared to a caloric restriction in reducing intra- abdominal fat and also not in reducing subcutaneous abdominal fat [10–12]. Others found a dose-response effect of volume of aerobic exercise on subcutaneous abdominal fat, but not intra-abdominal fat [13].

The Sex Hormones And Physical Exercise (SHAPE)-2 study was primarily designed to investigate the effect of equivalent weight loss with or without exercise on biomarkers of postmenopausal breast cancer risk. Results on sex hormones and inflammatory factors and adipokines are reported elsewhere [14, 15].

In this paper, we aim to address the following question: Does weight loss induced mainly by exercise in combination with a small caloric restriction, reduce (intra-)abdominal fat to a larger extent than equivalent weight loss induced by diet only?

## Methods

### Design and study population

The present study is a secondary analysis of the SHAPE-2 study, a randomized controlled trial in 243 healthy overweight to obese postmenopausal women.

The primary aim of the SHAPE-2 study was to assess whether exercise had additional beneficial effects to weight loss on biomarkers for breast cancer, since previous studies had shown that only in women who lost weight, biomarkers changed in the good direction. Therefore, in the SHAPE-2 study, we aimed to obtain an equal weight loss in both intervention arms (the weight loss was achieved by caloric restriction only; or by exercise and a small caloric restriction) [16].

The study ran from February 2012 until June 2013, in the Netherlands. Details of the study design are reported elsewhere [16, 17]. The ethical committee of the University Medical Center Utrecht approved the study (No. 11/465); and all study subjects provided written informed consent. Furthermore, this study adheres to CONSORT guidelines.

Recruitment was mainly performed by mass mailings sent to a random sample of women aged 50–69 years drawn from female inhabitants of eight municipalities in the Netherlands. Furthermore, we recruited via media attention. Eligible women were postmenopausal; overweight/obese (BMI 25–35 kg/m^2^); insufficiently physically active (< 2 h/week of moderate to vigorous activity); not using exogenous (sex) hormones; and not diagnosed with diabetes or cancer. Postmenopausal state was self-reported and defined as natural cessation of menses for at least 12 months, or in case of hysterectomy: aged 55+ and likely to be postmenopausal based on medical history.

### Interventions

The study started for all women with a 4–6 week run-in period in which a personalized standardized diet was prescribed conforming to the Dutch National Guidelines for a Healthy diet [18] i.e. 50–60% carbohydrate, 15–20% protein and 20–35% fat, maximum of 1 alcoholic drink per day; > 25 g of fibres per day; 200 g vegetables, 2 servings of fruit etc. The energy content of the standardized diet is determined using the individual’s habitual energy intake (dietary history), body weight history and a calculated estimate using the Harris & Benedict formula [19] multiplied by an estimate of their physical activity level. The run in period is designed to obtain a comparable macronutrient intake among participants, stabilise body weight, check the estimated energy requirements and evaluate protocol adherence. Hereafter, baseline measurements were taken and study participants were randomised, stratified for municipality, to a diet group (*n* = 97), exercise plus diet group (*n* = 98) or control group (*n* = 48). The randomisation software generated random sequences within block sizes of five (ratio interventions versus control; 2:2:1).

Both intervention programmes aimed to lose 5–6 kg of body weight, in 10–14 weeks’ time. Dietitians and physiotherapists supervised the weight loss interventions and were established in each participating municipality. Weekly self-weighing was performed in both intervention groups to closely monitor body weight. In addition, supervised weighing was performed by the dietitian (each visit) and physiotherapist (weekly). If the weekly weight loss of a participant did not meet or exceed the 0.5 kg/week for 3 consecutive weeks, extra coaching was provided to adapt the participants’ diet or exercise level. If the weight loss goal was reached, or after 14 weeks maximum, a weight maintenance period started (2–6 weeks) wherein with dietary adaptations energy intake and expenditure were balanced. The maintenance period aimed to stabilise body weight in order to obtain stable sex hormone levels. The control group was instructed to maintain a stable weight during the whole intervention period. They were offered an alternative weight loss programme after the study period.

Participants randomised in the diet group were prescribed a diet with a caloric restriction of 3500 kcal/week as compared to their estimated needs and habitual intake (i.e., standardised diet during the run-in period). They were instructed to maintain their habitual physical activity level.

Participants in the exercise plus diet group followed a 4-h/week exercise programme (energy expenditure of approximately 2530 kcal/week). The exercise programme included two 60-min group sessions of combined strength and endurance training conducted by physiotherapists and two 60-min Nordic walking (brisk walking with poles) sessions per week. Group sessions consisted of 25 min of strength training, 20–25 min of endurance training and 5–10 min warm ups and cool downs. During exercising, heart rate monitors were worn. Training intensity increased gradually during the study (for endurance training: from 60 to 90% of the heart rate reserve (HRR)), for strength training: based on repetition maximum tests). Nordic walking sessions were performed at 60–65% HRR. Nordic Walking instructor supervised classes were organised and women were strongly encouraged to attend these. For feasibility reasons, participants were also allowed to perform these sessions on their own. In this case, home training sessions should be filled in, in provided exercise logs and were checked by the physiotherapists. On top of the exercise programme, women were instructed to increase their daily energy expenditure by for example taking the bike for shopping and by climbing stairs. For specific details of the training programme, we refer to the design-paper of the SHAPE-2 study [16].

The exercise intervention was combined with a small caloric intake restriction of 1750 kcal/week to ensure the 5–6 kg weight loss in a short time frame. However, the emphasis was on exercise. The total targeted daily energy deficit in the exercise plus diet group (2530 kcal/week by exercise + 1750 kcal/week by diet = 4280 kcal/week) was larger than in the diet group (3500 kcal/week). This decision was taken since reduce and maintain body weight by exercise only in obese and untrained women is a long term process and a goal hard to attain. Several compensatory mechanisms, that are both mentally and physically (e.g. gain in muscle mass), unable adequate weight loss. Therefore, since the aim was an equal weight loss in both intervention groups, we decided to study the effect of exercise in combination with a slight caloric restriction [16].

Potential changes in weight, exercise and eating pattern in all three groups were monitored by phone calls by the study team or dietician. Additionally, 3-day food diaries were taken in the run-in period, after 4 weeks intervention and in the maintenance period. See also Table 1 for a schematic overview of the study.

**Table 1:** Overview study programme, contact moments and measurements

	RUN IN PHASE						INTERVENTION PHASE														MAINTENANCE	
week	−6	−5	−4	−3	−2	−1	1	2	3	4	5	6	7	8	9	10	11	12	13	14	15	16
Diet group																						
-diet composition	BASELINE DIET						ENERGY RESTRICTED DIET (−500 kcal/day)															
-dietary counselling^a^	F		T		T		F	G	T	G	T	G	T		T	G	T			F, G		T
Exercise plus diet group																						
-diet composition	BASELINE DIET						ENERGY RESTRICTED DIET (− 250 kcal/day)															
-dietary counselling^a^	F		T		T		F		T		T		T		T		T			F		T
-exercise programme^b^							AEROBIC AND STRENGTH TRAINING (4 h/week)															
							E	E	E	E	E	E	E	E	E	E	E	E	E	E	E	E
Control group																						
-diet composition	BASELINE DIET						BASELINE DIET															
-dietary counselling^a^	F		T		T		T				T											T
MEASUREMENTS^c^																						
-Habitual dietary intake (dietary history)	X																					
-Actual dietary intake (3-day food records)				X						X											X	
-Habitual physical activity (questionnaires)							X															X
-Actual physical activity (accelerometer)	X																				X	
-Anthropometrics (weight, waist, hip circumference); body composition (DEXA); visceral and subcutaneous abdominal fat (MRI); fitness (maximal exercise test); blood pressure; sex hormones (blood sampling)							X															X

^a^dietary counselling: F = face-to-face individual counselling; T = individual counselling by telephone; G = group counselling session

^b^ exercise programme: E = group fitness and Nordic walking, 4 h per week

^**c**^ measurements: X = all participants

### Outcome measurements

At baseline and after 16 weeks measurements were taken. Abdominal fat, including subcutaneous abdominal adipose tissue (SAAT) and intra-abdominal adipose tissue (IAAT), was determined by magnetic resonance imaging (MRI, Philips, Ingenia 1.5 Tesla) with the use of the three-point IDEAL Dixon method [20]. Of each individual the stack of fat weighted MRI images were selected and uploaded to the software programme ImageJ (https://imagej.net/Welcome) where the images could be viewed in the sagittal plane and slices on level L2-L3, L3-L4 and L4-L5 were selected. Hereafter, the fat weighted images of the single slices were selected and uploaded to the semi-automatic HippoFat software [21] (Additional file 1: Figure S1a). First, a line is drawn manually around the subcutaneous fat (Additional file 1: Figure S1b), to create a region of interest. Second, the software determines the area in that region that contains non-black pixels, the line is adjusted and an area is given in cm2. If necessary, the line can be adjusted manually again where after the area will be recalculated*.* Third, to determine the amount of visceral fat, a line is drawn manually around the intra-abdominal space to create a region of interest (Additional file 1: Figure S1c). The vertebrae and psoas muscles were left out of this region. The HippoFat software determines a curve that fits the grey values of the visceral fat. On the screen, all pixels that are included are coloured red (Additional file 1: Figure S1d) and the curve is adjusted manually based on visual checking, so that the visceral fat is covered properly. An area in cm2 is given automatically. The measured cm^2^ of SAAT and IAAT were averaged for the three slices. SAAT and IAAT were summed to obtain total abdominal adipose tissue (TAAT). Two trained researchers (WvG and MS) performed the fat quantifications. Baseline and follow-up images of different intervertebral levels of one study participant were analysed by the same researcher. Intra- and inter-class correlation coefficients for both researchers were > 0.99 for SAAT and > 0.97 for IAAT.

Total body fat and lean mass were assessed by whole body dual energy X-ray absorptiometry (DEXA, Lunar Prodigy). Height and body weight are measured wearing only light clothes and no shoes. Cardiorespiratory fitness (VO_2peak_) was measured by a maximal cycle exercise test with respiratory gas analysis using a ramp protocol.

To assess the level of habitual physical activity, the SQUASH questionnaire was used [22].

### Statistical analyses

Based on the study of Ross et al. [23] (in their obese study population: mean total intra-abdominal fat was 2.3 kg SD 0.8 kg), we calculated that at least 81 subjects are needed to demonstrate a difference of 0.2 kg intra-abdominal fat (Alpha = 0.05; Beta = 0.20.) between the ‘diet’ and ‘exercise plus diet’ group. The sample size of the control group is smaller, since the expected difference in fat loss with the interventions groups is larger.

To investigate the effects of equivalent weight loss, with or without exercise on intra- and subcutaneous abdominal fat tissue, linear regression was used to estimate the between-group differences for TAAT, SAAT and IAAT, adjusted for baseline fat measure. Outcomes of complete cases (women with measurements at both baseline and follow-up) are presented, since complete case analyses with covariate adjustment yield unbiased estimates in RCTs with missing outcome data [24].

All statistical procedures were performed using SPSS software version 20.

## Results

**Fig. 1:**
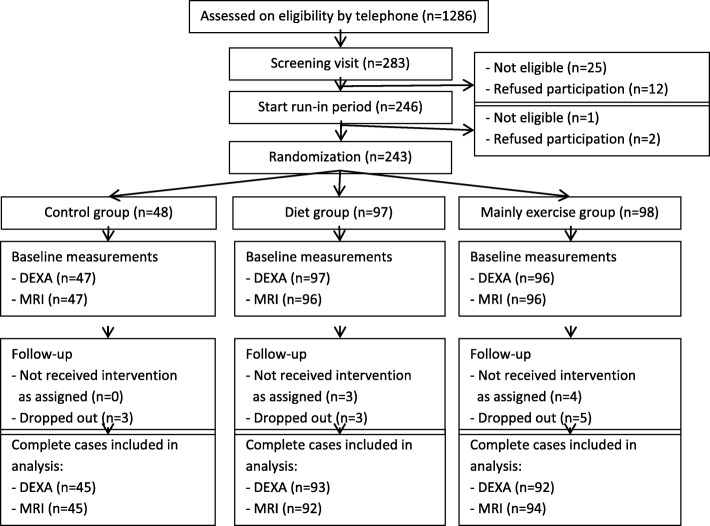
Flow chart of the inclusion, random assignment, and follow-up of the SHAPE-2 study participants. *Legend: The SHAPE-2 study is a three-armed RCT conducted in 2012–2013 in the Netherlands*

All three study groups were comparable for the main baseline characteristics (Table 2). Women were on average 60 years old, with a BMI of 29.2 kg/m^2^ and a body fat percentage of 44%. In total, 232 (95.5%) of all 243 women completed the study (Fig. 1).The number of women with complete case data on abdominal fat were 92 (95%) in the diet group, 94 (96%) in the exercise plus diet group and 45 (94%) in the control group.

**Table 2:** Baseline characteristics of the SHAPE-2 study population

	Control group (n = 48)	Diet group (n = 97)	Exercise+diet group (n = 98)
	*Mean (standard deviation)*		
Age (years)	60.0 (4.9)	60.5 (4.6)	59.5 (4.9)
Time since last menses (years)	11.4 (7.8)	10.7 (6.1)	10.9 (7.7)
Education^a^, number (%)			
Low	15 (31.3%)	27 (27.8%)	33 (33.6%)
Middle	15 (31.3%)	27 (27.8%)	20 (20.4%)
High	18 (37.5%)	42 (43.3%)	44 (44.9%)
First degree family member with breast cancer, number (%)	9 (18.8%)	23 (23.7%)	24 (24.5%)
VO2peak, relative (mL/kg/min)	22.1 (4.7)	21.9 (4.0)	21.8 (3.7)
Weight (kg)	80.9 (10.0)	80.0 (8.6)	80.4 (9.0)
BMI (kg/m^2^)	29.5 (2.6)	29.3 (2.5)	29.0 (2.9)
Body composition by DEXA			
Body fat percentage (%)	43.6 (5.0)	44.1 (3.8)	43.8 (4.0)
Total body fat (kg)	34.2 (7.4)	33.9 (5.7)	33.9 (6.2)
Lean mass (kg)	43.4 (3.9)	42.7 (4.0)	43.1 (4.1)
SQUASH moderate and vigorous activity^b^ (min/week) Dietary composition (%)	270 (120–495)	184 (115–420)	248 (90–465)
Fat	*32*	*32*	*34*
Carbohydrates	*45*	*44*	*43*
Protein	17	19	18
Alcohol	3	3	3
Undefined	3	2	2

Available baseline data: family history of breast cancer, *n* = 241 (99.2%); DEXA scan *n* = 240 (98.8%); MRI scan *n* = 239 (98.4%); VO_2peak_ *n* = 237 (97.5%); All other data was available for all women (*n* = 243)

^a^ Education, low: primary school and technical/professional school. Middle: college degree. High: university degree

^b^ Based on the SQUASH physical activity questionnaire, activities performed ≥4 METs

*BMI* Body mass index, *TAAT* Total abdominal adipose tissue, *SAAT* Subcutaneous abdominal adipose tissue, *IAAT* Intra-abdominal adipose tissue

The SHAPE-2 study is a three-armed RCT conducted in 2012–2013 in the Netherlands

There was a good compliance to the weight loss interventions. The median number of diet-group sessions attended was four (out of five offered) and 70% of women attended at least four sessions. All women attended the first individual face-to-face meeting with the dietitian for the standardised diet prescription in the run-in period. The second and third face-to-face contacts for prescriptions of the intervention diet and maintenance diet were attended by 98.4 and 91.4%, respectively. All other women received dietary prescriptions per mail which were discussed by telephone. The composition in macronutrients at study end was comparable to the composition at baseline.

The median attendance rate for the group exercise sessions in the exercise plus diet group was 81% and for the Nordic walking training hours it was 88%.

The outcomes on body composition and fitness of the SHAPE-2 trial were published elsewhere [15]. In short, both groups reached the weight loss goal. Participants in the diet group lost − 4.9 kg (− 6.1%) and − 5.5 kg (− 6.9%) in the exercise plus diet group. There was a decrease in body fat percentage, as compared to the control group, of − 2.8% (95% Confidence Interval (CI) -3.5;-2.1) with diet, and − 4.4% (95%CI -5.1;-3.7) with exercise plus diet. The decrease in fat percentage was significantly greater in the exercise plus diet group, compared with diet (difference of − 1.56, 95%CI -2.14;-0.98). Lean mass was preserved with exercise plus diet (+ 0.02 kg, 95%CI -0.42; 0.46), and decreased with diet (− 0.71 kg, 95%CI -1.14;-0.23), versus control. The level of moderate to vigorous physical activity, based on the SQUASH questionnaire, increased in the exercise plus diet group by 222 min/week (95% CI 43;401) compared to control and by 304 min/week (95% CI 158;451) compared to the diet group. VO_2peak_ increased significantly in women in the exercise plus diet group by 198 mL/min (95% CI 137; 260) versus control and by 32.0 ml/min (− 29.9; 93.8) versus diet.

Table 3 shows the baseline and end of study (i.e., 16 weeks) means and 95% CI of TAAT, SAAT and IAAT in the three study groups, and the within and between group changes. From baseline to end of study, fat measures remained stable in the control group and reduced significantly in both intervention groups. Reductions were approximately 12% in the diet group and 15–16% in the exercise plus diet group.

**Table 3:** Baseline and 16-week differences in body fat between study groups and treatment effects

Type of abdominal adipose tissue	Baseline mean	16 weeks mean	% Within group 16 weeks change	Within group 16 weeks change (95% CI)	P-value^a^	Between group 16 weeks change intervention vs control (95% CI)	P-value^b^	Between group 16 weeks change Exercise vs Diet (95% CI)	P-value^c^
Total (TAAT, cm^2^)									
Control	461	466	1.1%	4.9 (−4.1 to 13.9)	0.282				
Diet	454	398	−12.3%	−55.8 (−63.8 to − 47.8)	< 0.001	−60.8 (− 74.3 to − 47.3)	< 0.001		
Exercise+diet	449	379	−15.7%	−70.4 (−78.5 to −62.2)	< 0.001	−75.4 (−88.8 to −61.9)	< 0.001	− 14.6 (−25.5 to −3.7)	0.009
Subcutaneous (SAAT,cm^2^)									
Control	311	315	1.2%	3.6 (−3.5 to 10.7)	0.308				
Diet	312	273	−12.5%	− 38.9 (−44.7 to − 33.0)	< 0.001	−42.4 (−52.6 to − 32.3)	< 0.001		
Exercise+diet	306	257	−16.0%	−49.1 (−55.4 to − 42.8)	< 0.001	−53.0 (−63.1 to − 42.9)	< 0.001	−10.6 (− 18.7 to − 2.4)	0.011
Intra-abdominal (IAAT,cm^2^)									
Control	150	152	1.6%	2.4 (−3.4 o 8.2)	0.406				
Diet	142	125	−12.0%	−17.0 (−20.9 to −13.0)	< 0.001	−20.1 (− 26.6 to −13.75)	< 0.001		
Exercise+diet	143	122	−14.6%	−20.9 (− 24.4 to −17.4)	< 0.001	−24.0 (−30.4 to − 17.6)	< 0.001	− 3.8 (−9.0 to 1.3)	0.145

*TAAT* Total abdominal adipose tissue, *SAAT* Subcutaneous abdominal adipose tissue, *IAAT* Intra-abdominal adipose tissue a. A *P*-value of < 0.05 was considered significant for the within group change

b. A *P*-value of < 0.025 was considered significant for the comparison of both intervention groups versus control. c. A *P*-value of < 0.05 was considered significant for the comparison exercise versus diet

Between-group changes showed that compared with control, TAAT, SAAT and IAAT decreased significantly in both intervention groups (*p* < 0.001). The decreases in TAAT and SAAT were significantly larger in the exercise plus diet group compared with diet: differences of − 15 cm2 (95%CI -26;-4) for TAAT and − 11 cm^2^ (95%CI -19;-2) for SAAT. IAAT decreased slightly more in the exercise plus diet group (− 24 cm^2^, 95%CI -30;-18) than in the diet group (− 20 cm^2^, 95%CI -27;-14), compared with control, although not statistically significant. The between group difference in IAAT between exercise plus diet versus diet was − 4 cm2 (95%CI -9; 1.3).

## Discussion

We found that 6–7% weight loss by either diet only or diet combined with exercise in overweight to obese postmenopausal women reduced both subcutaneous and intra-abdominal body fat. When weight loss was induced by a combination of diet and exercise, slightly larger declines in subcutaneous abdominal fat occurred, however, also in body weight reduction. Intra-abdominal fat loss was not statistically significantly larger in the exercise plus diet group.

Other weight loss trials with diet and exercise interventions in postmenopausal women also found a decline in subcutaneous and intra-abdominal fat, but they did not find significant stronger effects for weight loss induced by a combination of caloric restriction and exercise [10–12]. In one trial, 112 postmenopausal overweight and obese women were randomised to diet only (i) or diet with either moderate (ii) or vigorous (iii) intensity exercise during 20 weeks [11]. Although the caloric deficit was similar between all groups, comparable to our study, weight loss differed slightly between the study groups. The diet only group lost on average 11.8 kg compared to 12.2 kg and 12.3 kg in the diet plus moderate or vigorous exercise, respectively. Abdominal subcutaneous fat decreased statistically significantly by 18% and intra-abdominal fat by 26% from baseline. Intra-abdominal fat loss was largest in the diet plus vigorous exercise group and subcutaneous abdominal fat loss was largest in the diet only group, but both not significantly different from diet only. The mean amount of weight loss was larger than in our trial (− 12 kg versus − 5.5 kg), probably explaining the larger percentages of fat losses. In another RCT in 77 obese postmenopausal women, six months of diet with or without aerobic exercise induced approximately 7 kg of weight loss (7 kg diet only and 7.2 kg diet plus exercise) [12]. Again, subcutaneous and intra-abdominal fat declined significantly and to a similar extent from baseline (both by − 15%) in both weight loss groups. Similar results were found in a small trial of 34 obese postmenopausal women wherein the effects of weight loss and resistance training were investigated over 16 weeks [10]. Diet (− 5.2 kg) and diet with resistance training (− 7.4 kg) led to comparable changes in subcutaneous abdominal fat (both − 18%) and intra-abdominal fat (both − 22%).

We observed levels of subcutaneous abdominal fat loss in the same range compared to above studies (− 16% for exercise plus diet induced; − 12.5% for diet induced weight loss). An explanation for the fact that we observed slightly greater loss of subcutaneous abdominal fat in the exercise plus diet group compared with diet only group, could be that women in our trial were prescribed four hours/week of exercise, instead of two or three hours/week as in the above trials. A dose response effect of exercise on subcutaneous abdominal fat loss in postmenopausal women is supported by the BETA trial [13]. In the BETA trial, two groups performed aerobic exercise for 150 and 300 min and lost 1.8 and 2.5 kg of body weight, respectively. There was no difference in intra-abdominal fat loss between the groups. In the high-dose group, subcutaneous fat was decreased more than in the low-dose group, but this might also (partly) be explained by the 0.7 kg more loss of body weight. This might also (partly) explain our results; women in the exercise plus diet group in our trial lost 0.6 kg more body weight than the diet group.

In our study, intra-abdominal fat loss was not significantly larger in the exercise plus diet group compared to the diet only group, which is in accordance with the trials mentioned above. We expected that weight loss mainly by exercise would affect intra-abdominal fat more than equivalent weight loss by diet only, because exercise causes lipolytic hormones to be secreted, facilitating greater post-exercise energy expenditure and fat oxidation [25, 26]. It seems, however, that combining diet with exercise, or different intensities of exercise have no other effect on intra-abdominal fat loss compared to diet alone, if the caloric deficit is (almost) the same. This finding is comparable to a recent review by Merlotti et al. [27] which evaluated the amount of intra-abdominal and subcutaneous fat loss with all available strategies (diet and exercise, weight-loss promoting agents and bariatric surgery). They conclude that there is no evidence of a weight loss intervention that preferentially targets intra-abdominal fat mass. The meta-analysis showed that, regardless of the weight loss strategy used, the absolute amount of subcutaneous abdominal fat loss is greater than intra-abdominal fat loss and percentage intra-abdominal fat loss is greater than percentage subcutaneous abdominal fat loss, however, no differences between different weight loss strategies were found [27]. Although exercise does not result in specific intra-abdominal fat loss, we found that exercise positively affects body composition (preserved or more lean body mass and less total body fat) and increases the aerobic capacity [15], and has, therefore, beneficial effects compared to other weight loss strategies. So far, a specific training intensity or modus that can specifically target intra-abdominal fat in postmenopausal women has not been identified.

Strengths of our trial include the large sample size and the randomised design with two different interventions aiming for equivalent weight loss.

Moreover, the use of MRI, a precise and valid standard method to measure changes in abdominal fat, distinguishes our study from the previous literature [28]. Additionally, instead of a single slice analysis which is most often used, fat quantifications were derived from three MRI slices at different abdominal levels.

Despite the aim for equivalent weight loss, the exercise plus diet group experienced a 0.6 kg greater weight loss than the diet group; which is a limitation and may have affected the results. Furthermore, a longer intervention or follow-up duration might be necessary to show potential effects of exercise-induced weight loss above weight loss by diet only on intra-abdominal fat loss.

## Conclusion

We found that a 6–7% weight loss in healthy and overweight-to-obese postmenopausal women led to a decrease in both intra-abdominal and abdominal subcutaneous fat. Weight loss when exercise is combined with a small caloric restriction, seems to produce greater changes in subcutaneous abdominal fat, but not in intra-abdominal fat, as compared to weight loss induced by caloric restriction only. This effect on subcutaneous fat might partly be explained by the slightly greater amount of weight loss in the exercise plus diet group.

## Additional file

Additional file 1: **Figure S1.** Illustration of the abdominal fat assessment method using the semi-automated HippoFat software. **a** The fat weighted images of a single MRI slice. **b** As a first step, a line is drawn manually around the subcutaneous fat volume to create a region of interest. Thereafter, the software determines the area that contains non-black pixels in this region of interest, the line is adjusted and an area is given in cm^2^. If necessary, the line can be adjusted manually again and the area can be recalculated. **c** To determine the amount of visceral fat, a line is drawn manually around the intra-abdominal space to create a region of interest. The vertebrae and psoas muscles were left out of this region. **d** The HippoFat software determines a curve that fits the grey values of the visceral fat. On the screen, all pixels that are included are coloured red and the curve is adjusted manually based on visual checking, so that the visceral fat is covered properly. An area in cm^2^ is given automatically by the software. (DOCX 521 kb)

## Abbreviations

BMI: Body Mass Index
DEXA: Dual Energy X-ray Absorptiometry
HRR: Heart Rate Reserve
IAAT: Intra-abdominal Adipose Tissue
KCAL: Kilocalories
MRI: Magnetic Resonance Imaging
RCT: Randomised Controlled Trial
SAAT: Subcutaneous Abdominal Adipose Tissue
TAAT: Total Abdominal Adipose Tissue
