# Supplementary figures and images for: Effect of diet with or without exercise on abdominal fat in postmenopausal women – a randomised trial
Source: BMC Public Health. 2019 Feb 11;19:174. doi: 10.1186/s12889-019-6510-1 (PMC6371569; doi:10.1186/s12889-019-6510-1)

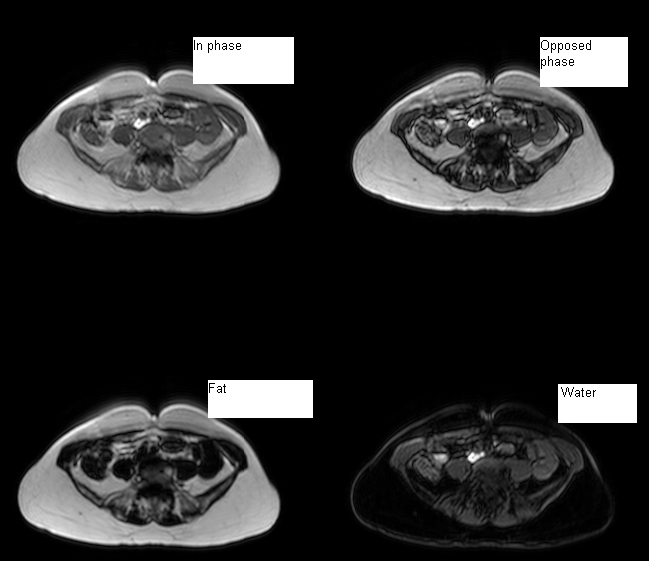


Figure 1a
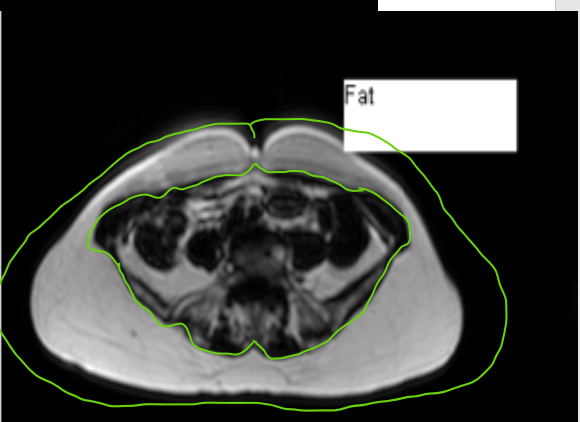


Figure 1b


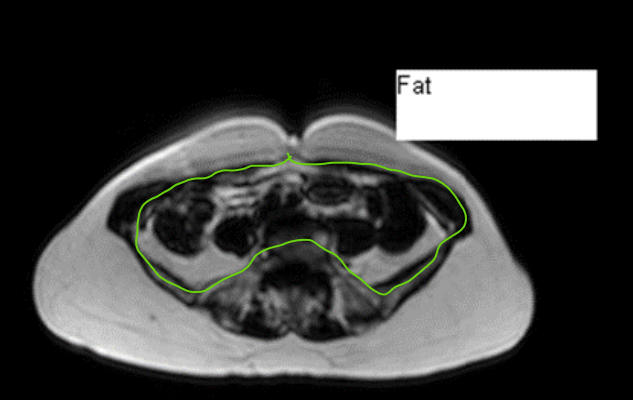


Figure 1c


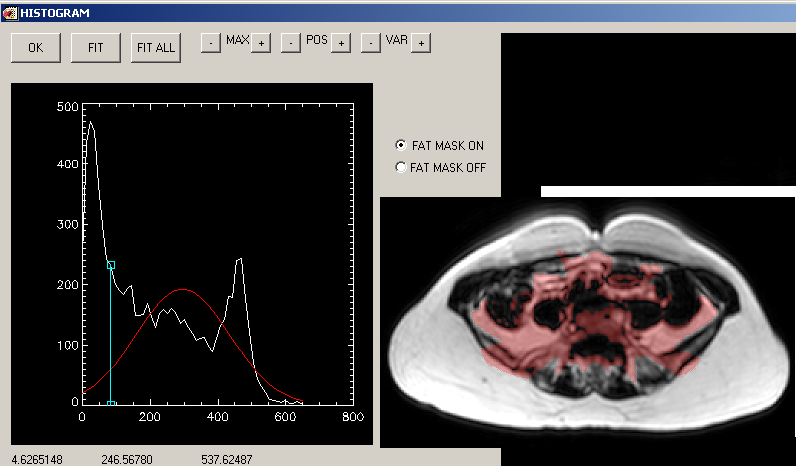


Figure 1d

Supplement: Supplementary file 1 — Figure S1. Illustration of the abdominal fat assessment method using the semi-automated HippoFat software. a The fat weighted images of a single MRI slice. b As a first step, a line is drawn manually around the subcutaneous fat volume to create a region of interest. Thereafter, the software determines the area that contains non-black pixels in this region of interest, the line is adjusted and an area is given in cm2. If necessary, the line can be adjusted manually again and the area can be recalculated. c To determine the amount of visceral fat, a line is drawn manually around the intra-abdominal space to create a region of interest. The vertebrae and psoas muscles were left out of this region. d The HippoFat software determines a curve that fits the grey values of the visceral fat. On the screen, all pixels that are included are coloured red and the curve is adjusted manually based on visual checking, so that the visceral fat is covered properly. An area in cm2 is given automatically by the software. (DOCX 521 kb) [file 12889_2019_6510_MOESM1_ESM.docx]
